# Flow Rate and Interference Studies for Copper Binding to a Silica-Immobilized Humin Polymer Matrix: Column and Batch Experiments

**DOI:** 10.1155/BCA.2005.1

**Published:** 2005

**Authors:** Jorge L. Gardea-Torresdey, Carolina Contreras, Guadalupe de la Rosa, Jose R. Peralta-Videa

**Affiliations:** 1 Chemistry Department University of Texas at El Paso El Paso TX 79968 USA; 2 Environmental Science and Engineering Ph.D. Program University of Texas at El Paso El Paso TX 79968 USA

**Keywords:** humin-silica biopolymer, packed-bed column, heavy metals, removal, copper, hard cations

## Abstract

Batch and column experiments were performed to determine the Cu(II) binding capacity of silica-immobilized humin biomass. For column studies, 500 bed volumes of a 0.1 mM Cu(II) solution were passed through humin packed columns at the flow rates of 1, 1.5, 2, and 3 mL/min. The biopolymer showed an average Cu binding capacity of 12 ± 1.5 mg/g and a Cu recovery of about 96.5 % ± 1.5. The breakthrough points for Cu(II) alone were approximately 420, 390, 385, and 300 bed volumes for the flow rates of 1, 1.5, 2 and 3 mL/min, respectively. The interference studies demonstrated that at low concentrations, the hard cations Ca(II) and Mg(II) did not seem to represent a major interference on Cu(II) binding to the humin biopolymer. The selectivity showed by this biopolymer was Cu(II)>Ca(II)>Mg(II). On the other hand, batch experiments showed that Ca(II) + Mg(II) at 100mM each reduced the Cu(II) binding to 73 %. However, 1000 mM concentrations of Ca(II) and Mg(II), separately and in mixture, reduced the Cu(II) binding to 47 %, 44 % and 31 %, respectively. The results of this study showed that immobilized humin in a silica matrix could represent an inexpensive bio-source for Cu removal from contaminated water, even in the presence of low concentrations of the hard cations Ca(II) and Mg(II).


### Flow Rate and Interference Studies for Copper Binding to a Silica-Immobilized Humin Polymer Matrix: Column and Batch Experiments

Jorge L. Gardea-Torresdey[1,2]*, Carolina Contreras[1], Guadalupe de la Rosa[2], Jose R. Peralta-Videa[1]

[1]*Chemistry Department and* [2]*Environmental Science and Engineering Ph.D. Program*
*University of Texas at El Paso, El Paso, TX 79968, U.S.A.*

### ABSTRACT

Batch and column experiments were performed to determine the Cu(II) binding capacity of silica-immobilized humin biomass. For column studies, 500 bed volumes of a 0.1mM Cu(II) solution were passed through humin packed columns at the flow rates of 1, 1.5, 2, and 3 mL/min. The biopolymer showed an average Cu binding capacity of 12 ± 1.5 mg/g and a Cu recovery of about 96.5 % ± 1.5. The breakthrough points for Cu(II) alone were approximately 420, 390, 385, and 300 bed volumes for the flow rates of 1, 1.5, 2 and 3 mL/min, respectively. The interference studies demonstrated that at low concentrations, the hard cations Ca(II) and Mg(II) did not seem to represent a major interference on Cu(II) binding to the humin biopolymer. The selectivity showed by this biopolymer was Cu(II)>Ca(II)>Mg(II). On the other hand, batch experiments showed that Ca(II) + Mg(II) at 100mM each reduced the Cu(II) binding to 73 %. However, 1000 mM concentrations of Ca(II) and Mg(II), separately and in mixture, reduced the Cu(II) binding to 47 %, 44 % , and 31 %, respectively. The results of this study showed that immobilized humin in a silica matrix could represent an inexpensive bio-source for Cu removal from contaminated water, even in the presence of low concentrations of the hard cations Ca(II) and Mg(II).

**Keywords:** humin-silica biopolymer, packed-bed column, heavy metals, removal, copper, hard cations.

### INTRODUCTION

Humic substances (humic and fulvic acids and humin) have an important role in soil processes /1/. These substances consist of a complex mixture of molecules whose molecular weight varies from hundreds to over

*Corresponding author. Phone: +1 (915) 747 5359; fax: +1 (915) 747 5748;
E-mail: jgardea@utep.edu

300,000 g/mol /2/. Humin represents the humic fraction of the natural organic matter that cannot be extracted with organic solvents or diluted in basic or acidic solutions. Due to its high molecular weight and carbon content, humin can partly elude biodegradation and fossilize after deposition in hydromorphous environments /1/. Furthermore, humin is often the main form of organic matter in many types of soils /1/. It has been reported that humic substances can interact with metals via chelation, physical adsorption and cation exchange processes. However, the structure of these substances remains largely unknown /1,3/.

Heavy metals are common contaminants derived from industrial activities and considerable efforts have been made in order to remove these contaminants from soils and aqueous solutions /4,5/. However, scientists and engineers are still searching for more economical solutions to alleviate these problems. Although the use of biomass as heavy metal sorbent has been documented in the literature /4,6-13,17-19/, further investigation needs to be performed in order to understand the binding mechanisms of heavy metals to organic molecules. As an example, it has been found that peat moss contains polar functional groups such as ketones, aldehydes, acids and phenols that might be involved in the chemical binding of heavy metals /12/. Batch and column experiments have been performed with peat moss, activated carbon, plant biomass, and chitin to elucidate both the heavy metal binding ability of the biomass and the binding mechanisms /6-10/. Similarly, several researchers have reported on Cu binding to humic fractions /5-7,9/; however, there is not enough information about the performance of these biomasses under flow conditions.

Although it has been demonstrated that different biomasses bind heavy metals, usually the biomass cannot be packed alone into a column because the small particles might clog the pores restricting the solution flow. However, if the biomass is immobilized in a polymer matrix, bigger particles can be obtained and packed into a column through which a flow rate can be achieved. In addition, the immobilized biomass offers the possibility to be used several times. The objective of this research was to evaluate the efficiency of native and immobilized humin biomass to remove Cu(II) ions from aqueous solutions under flow conditions. Additionally, experiments were performed to evaluate the recycling capacity of the column as well as their efficiency to adsorb Cu(II) in the presence of the interference cations Ca(II) and Mg(II).

### 2. MATERIALS AND METHODS

### 2.1 Extraction of humin

The procedure used in the present investigation to extract humin was previously described by Gardea-Torresdey *et al.* /13/. A fraction of 100 g of Canadian Sphagnum peat moss (Fisons Horticulture, Inc., Vancouver, B.C., Canada) was dried at 51°C for 72 h. The dried biomass was ground to a fine powder and sieved through an 80–mesh screen (0.177 mm). Afterwards, the powder was washed twice with 0.01M HCl and centrifuged for 5 minutes at 3000 rpm (Fisher Scientific, Marathon 6 K). Subsequently, 500 mL of 0.1M NaOH were added to the biomass and the pH of the solution was adjusted to 13.5 by the addition of 5M NaOH. The solution was stirred for 48 h and then centrifuged at 3000 rpm. The humin fraction precipitated and the humic acids remained in the supernatant. The humin fraction was washed twice with deionized (DI) water to eliminate the remaining alkalinity. Afterwards, it was freeze-dried on a Labconco freeze-dryer system (Freezone 4.5, Kansas City, MO) at –45°C and 69 X $10^{-3}$ Mbar pressure. The dried biomass was again ground and sieved through a 100-mesh screen (aprox.150 μm size particle).

### 2.2 Immobilization of humin biomass

The procedure followed for humin immobilization was similar to the one reported by Gardea-Torresdey *et al.* /8/. Twenty g of humin previously sieved through a 100-mesh screen (0.149 mm) were washed twice with 0.01M HCl and once with DI water. The washings were collected, evaporated and weighed to record any loss of biomass. Three hundred mL of 5 % $H_2SO_4$ were placed in a 2-L beaker and a solution of 6 % $Na_2SiO_3$ was added until a pH of 2 was reached. Under continuous stirring, the washed biomass was added to the $H_2SO_4$/ $Na_2SiO_3$ solution and allowed to equilibrate for approximately 15 min. Additional sodium silicate solution was added until a pH of 7 was reached and the polymer started forming. The polymer gel was washed with DI water until washings were negative for sulfate presence by using the $BaCl_2$ test (formation of $BaSO_4$ precipitate). The polymer was dried overnight in an oven at 60°C; then, the polymer was ground using a mortar and pestle and sieved to pass the 20-40-mesh size (approximately 0.841-0.354 mm).

### 2.3 Column study for Cu(II) adsorption by silica-immobilized humin

Column experiments were performed at pH 5 ± 0.1, since de la Rosa *et al.* /6/ reported that this is the optimal pH for Cu(II) binding to humin. The columns were packed using 3 mL of silica-immobilized humin (this volume of biomass was considered as one bed volume). After packing, the columns were washed with 0.01M HCl to discard any possible metal contamination. Subsequently, the columns were washed with DI water previously adjusted to pH 5 ± 0.1 until the washings were shown to have the same pH value. To determine the optimal flow rate for Cu binding to the silica-immobilized humin, 500 bed volumes of a 0.1mM Cu(II) solution were passed through different columns using the following flow rates: 1, 1.5, 2, and 3 mL/min. The effluents of each column were collected and the metal content was determined with a Flame Atomic Absorption Spectrometer (FAAS) (Perkin Elmer model 3110). Three cycles were run on each column to determine the capacity of the polymer after the corresponding saturation and stripping cycle. In order to recover the metal ions adsorbed to the column, 30 bed volumes of 0.1M HCl were passed and the corresponding effluents were collected and analyzed by FAAS to quantify Cu ions and the percent of recovery. After each cycle, the columns were washed with DI water previously adjusted to pH 5 on inverted flow, in order to destroy any preference channels that might have been formed. The washing was stopped when the column reached the pH of 5 ± 0.1.

### 2.4 Batch experiments for the interference of Ca(II) and Mg(II) on Cu(II) binding to humin.

Table 1 shows the concentration of the solutions used in this study. The concentrations of Ca(II) and Mg(II) solutions varied from 0 mM to 1000 mM, while Cu(II) was kept at 0.1mM. The following compounds: $Ca(NO_3)_2$. $4H_2O$, $Mg(NO_3)_2$. $6H_2O$, and $Cu(NO_3)_2$ were used as cation sources. A portion of 500

mg of biomass was washed 3 times with 0.01M HCl and 3 times with double deionized water (DDI) to reduce any external source of Ca(II) and Mg(II). The biomass was resuspended in 100 mL of DDI water to obtain a final concentration of 5 mg/mL, which was adjusted to pH 5 ± 0.1 using either $HNO_3$ or NaOH. Subsequently, aliquots of 4 mL were transferred to 5 mL test tubes, centrifuged at 3000 rpm for 5 min and the supernatants were discarded. Afterwards, 4 mL of the Cu(II) solution adjusted to pH 5 ± 0.1 were added to the reaction tubes, placed on a rocker and allowed to react for 1 h. Finally, the tubes were centrifuged for 5 min at 3000 rpm, and the cations Ca, Mg, and Cu were analyzed in the supernatant using Inductively Coupled Plasma Optical Emission Spectroscopy (ICP/OES). Each treatment was replicated three times for statistical purposes.

**Table 1**

Solution concentrations used on batch experiments for Ca(II) and Mg(II) interference on Cu(II) binding to silica-immobilized humin. Solutions were adjusted to pH 5 ± 0.1.

| Metal | Concentration (mM) | | | | | | | | | |
|---|---|---|---|---|---|---|---|---|---|---|
| **Cu(II)** | | | | | 0.1 | | | | | |
| **Ca(II)** | 0 | 0.1 | 0.2 | 10 | 20 | 100 | 200 | 0.1 | 0.2 | 1000 |
| **Mg(II)** | 0 | 0.1 | 0.2 | 10 | 20 | 100 | 200 | 0.1 | 0.2 | 1000 |

### 2.5 Column studies for the interference of Ca(II) and Mg(II) on Cu(II) binding

Table 2 shows the metal mixtures and the concentrations of the solutions used for this experiment. The concentration of Cu(II) was maintained at 0.1 mM, while the solutions containing either Ca(II), Mg(II) or both were kept at a concentration of 1 mM. Three columns were packed as described in section 2.3, and 500 bed volumes of the corresponding solution were passed through each column. The flow rate used was 2 mL/min, and all the solutions were prepared using DDI water.

**Table 2**

Concentrations and mixtures of cations used in column studies for the interference of Ca(II) and Mg(II) on Cu(II) binding to the humin biopolymer.

| Column 1 | Column 2 | Column 3 |
|---|---|---|
| 0.1mM Cu(II) | 0.1mM Cu(II) | 0.1mM Cu(II) |
| 1mM Ca(II) | 1mM Mg(II) | 1mM Ca(II) |
| | | 1mM Mg(II) |

### 2.6 Metal Analyses

### *2.6.1 Metal analyses for batch experiments*

The metal analyses for batch experiments were performed using an ICP/OES Perkin-Elmer Optima 4300

DV with an AS-90 plus auto sampler rack. The following parameters were utilized: nebulizer flow 0.7 L/min, radio frequency power 1300 watts; sample introduction 1.45 mL/min; flush time 10 sec; delay time 60 sec; read time 10 sec; wash time 45 sec; replicates 3, and each sample was read three times. Standards were prepared from a 1000-ppm Cu(II) stock solution and diluted with 5% $HNO_3$. The stock solutions of Ca(II) and Mg(II) had a concentration of 2M diluted with 5% $HNO_3$. The blank and six points were used to obtain the calibration curve, and the correlation coefficients ($r^2$) were 0.999 or better.

#### *2.6.2 Metal analysis for column experiments*

As previously described, the copper analysis in column experiments was performed using FAAS. Six standards were used to obtain a calibration curve with a minimum correlation coefficient ($r^2$) of 0.99. Standards were prepared from a 1000-ppm Cu standard solution and diluted with 0.01M HCl. The readings were performed at 327.4 nm and an impact bead was used to improve the instrument sensitivity /14/. Samples were analyzed up to 500 bed volumes and the difference between the metal concentration in the control solution and the metal concentration found in the effluent was assumed to be the Cu bound to the column.

### 2.7 Environmental Scanning Electron Microscopy (ESEM)

The scanning electron microscopy technique was used to obtain information about the surface of the biomass with the polysilicate matrix support. The results obtained herein provided information about the interactions between the biomass and the metals before and after saturation and stripping. An environmental scanning electron microscope (ESEM) model 2020 was used to record micrographs of the particles and to analyze the surface composition of the polymer.

### 3. RESULTS AND DISCUSSIONS

### 3.1 Effect of flow rate on Cu(II) adsorption by a silica-immobilized humin column.

Column experiments were performed at pH 5 ± 0.1, since previous batch experiments demonstrated that humin extracted from Canadian Sphagnum peat moss showed its best Cu(II) binding capacity at this pH /6/. Table 3 shows the effect of four different flow rates on Cu(II) desorption from the humin biopolymer. This table shows that the percentage of Cu recovery was pretty much the same in the three cycles, regardless of the flow rate used. Similarly, the amounts of Cu bound to the biomass at the different flow rates showed little differences among them (Table 4). Table 3 also shows that the percentage of Cu recovery from the biomass under flow conditions had an average of 98 % for the 1 and 1.5 mL/min flow rates, but 97 and 94 % for the flow rates 2 and 3 mL/min, respectively. On the other hand, the column showed an average binding capacity of 11.9 ± 0.3 mg Cu/g of biomass (Table 4). The Cu binding capacity shown by silica-immobilized humin is larger that the binding capacity reported by other researchers. Kappor *et al.* /17/ reported that *Aspergillus niger* binds 2.09 mg Cu/g of biomass; Blanco *et al.* /18/ found that *Phormidium Laminosum* immobilized-biomass removed 10 mg/g Cu, while Johnson *et al.* /20/ reported that the maximum Cu binding capacity of peanut hull was 9 mg/g.

**Table 3**

Percentages of metal recovery and effect of flow rate on Cu(II) adsorption by silica-immobilized humin. After each saturation cycle, a solution of 0.1M HCl was used as the stripping agent. Data represent average ± standard error (SE).

| Flow rate (mL/min) | Cu Recovery (%) | | | |
|---|---|---|---|---|
| | 1st cycle | 2nd cycle | 3rd cycle | Average |
| 1 | 98 | 98 | 97.4 | 97.8 ± 0.16 |
| 1.5 | 98 | 97 | 101 | 98.6 ± 0.98 |
| 2 | 97 | 96.7 | 97.2 | 96.9 ± 0.21 |
| 3 | 95 | 94 | 94 | 94.3 ± 0.27 |

**Table 4**

Adsorption capacity of silica-immobilized humin for Cu(II) binding at different flow rates. Each saturation cycle was run with 500 bed volumes at pH 5 ± 0.1. Data are average ± standard error (SE).

| Flow rate (mL/min) | Cu Bound (mg/g) | | | |
|---|---|---|---|---|
| | 1st cycle | 2nd cycle | 3rd cycle | Average |
| 1 | 10.9 | 11.7 | 12 | 11.5 ± 0.08 |
| 1.5 | 11.6 | 13 | 13.8 | 12.8 ± 0.52 |
| 2 | 12 | 11 | 10.7 | 11.2 ± 0.31 |
| 3 | 10.8 | 12.8 | 12.8 | 12.1 ± 0.36 |

The breakthrough curves for Cu(II) at different flow rates are shown in Figures 1 to 4. Figure 1 shows that at the flow rate of 1 mL/min, Cu appears in the effluent after 420, 415, and 405 bed volumes in the first, second and third saturation cycles, respectively. Thus, in the third cycle the column showed a decrease in its Cu binding capacity of about 4%, which indicates the potential reusability of the column. Figure 2 shows the breakthrough curves for the 1.5 mL/min flow rate. As shown in this figure, in the first cycle Cu(II) appears in the effluent after 380 bed volumes (without saturation of the column). The Cu(II) concentration in the effluent at that point was only 0.6 mg/L, this means 5.44 mg/L less than the Cu concentration in the influent that was 6 mg/L. In this cycle, even at 450 bed volumes no saturation of the column was observed. In the second cycle the breakthrough point was detected after 360 bed volumes, and up to 500 bed volumes the Cu(II) concentration was around half the concentration of this metal in the fed solution. Finally, on the third cycle the breakthrough point appeared 50 bed volumes before as compared to the one observed in the second cycle. Figure 3 shows the breakthroughs obtained using the 2 mL/min flow rate. The breakthrough point for

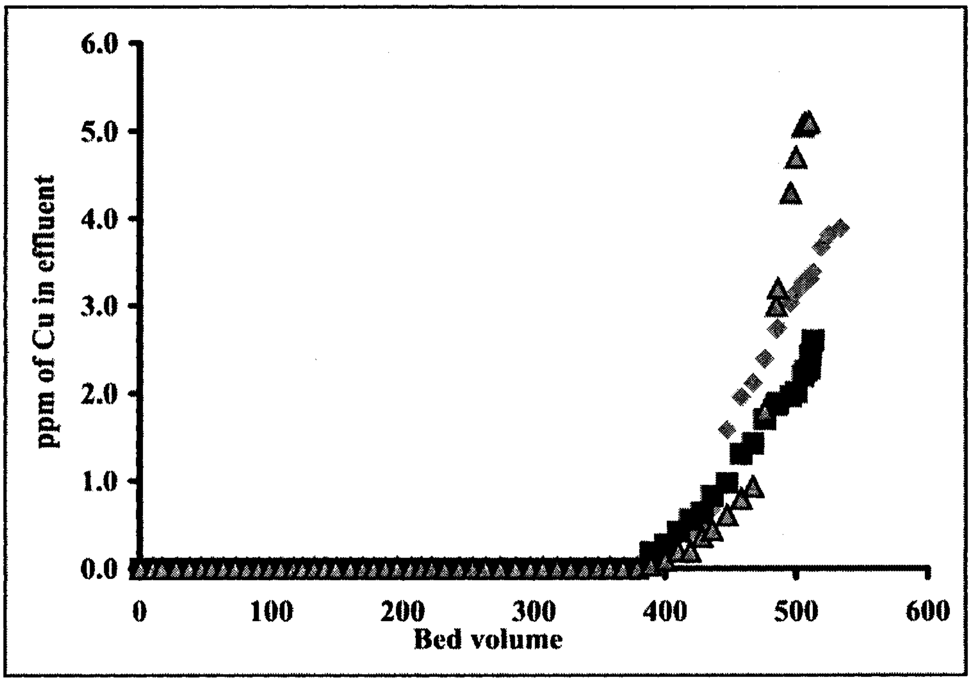

**Fig. 1:** Breakthrough curve for Cu(II) adsorption by the humin biopolymer. Flow rate used was 1 mL/min. The 0.1mM Cu(II) solution was adjusted at pH 5 ± 0.1.
◆ 1st cycle, ■ 2nd cycle, ▲ 3rd cycle.

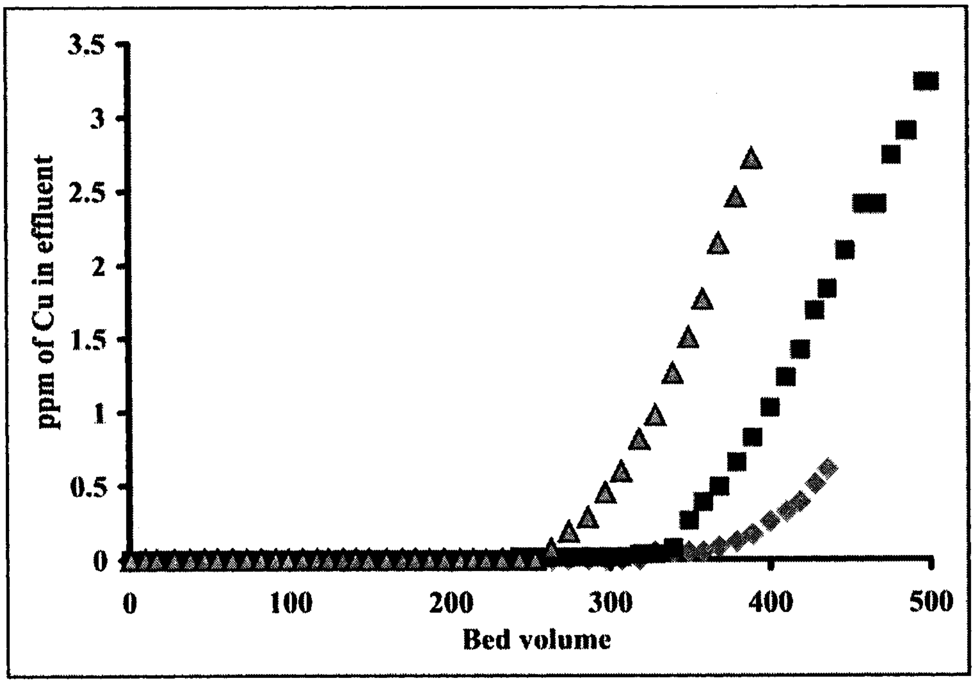

**Fig. 2:** Breakthrough curve for Cu(II) adsorption by the humin biopolymer. Flow rate used was 1.5 mL/min. The 0.1mM Cu(II) solution was adjusted at pH 5 ± 0.1.
◆ 1st cycle, ■ 2nd cycle, ▲ 3rd cycle.

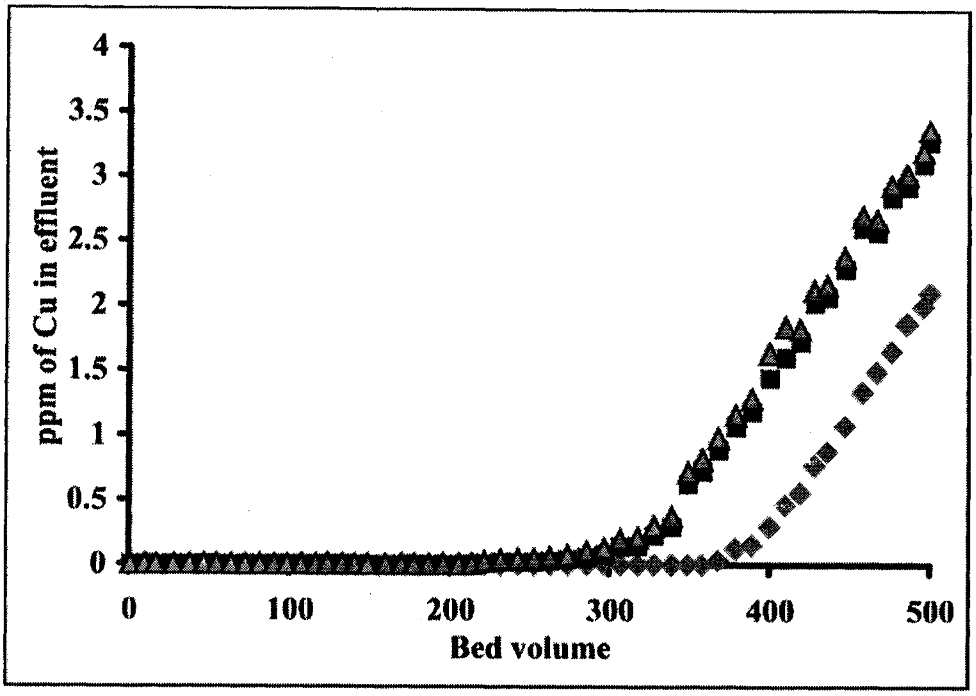

**Fig. 3:** Breakthrough curve for Cu(II) adsorption by the humin biopolymer. Flow rate used was 2 mL/min. The 0.1mM Cu(II) solution was adjusted at pH 5 ± 0.1.
◆ 1st cycle, ■ 2nd cycle, ▲ 3rd cycle.

the first cycle appeared at almost 400 bed volumes, decreasing 50 bed volumes in the second cycle. However, the breakthrough point was almost the same in the second and third cycles. It was also found that up to 500 bed volumes the column did not reach the saturation point in the three cycles, since the concentration of Cu in the influent was 4.8 mg/L but in the effluent was 3.1 mg/L. Similar results were found by de la Rosa *et al.* /19/ in experiments performed with the same biomass at this flow rate. Figure 4 displays the breakthrough curves for the 3-mL/min-flow rate. This figure shows that in the first and second cycle Cu appeared in the effluent solution after 280 bed volumes, but even after 500 bed volumes the column was not saturated. The Cu(II) concentration in the effluent were 2.4 and 3.4 ppm for the first and second cycle, respectively. These concentrations were, respectively, 3.6 and 2.4 less than the concentration in the fed solution that was 6 mg/L. In the third cycle the breakthrough point appeared at 230 bed volumes. Although Cu appeared earlier as compared to the first and second cycles, after 500 bed volumes the column was not saturated, since at this point the concentration of Cu(II) in the effluent was 4.4 mg/L. Based on these observations, it is possible to assume that the humin-immobilized biomass can be used for additional cycles.

It has been proposed that carboxyl groups may represent an important role in Cu binding /16/; thus, by lowering the pH, it is possible to protonate the copper-carboxylate moieties and the metal ions may be put back into the solution. Based on this, Cu was desorbed after each saturation cycle by using 24 bed volumes of 0.1 M HCl. The results are given in Table 3. According to the data, Cu was recovered in a high percentage in each of the three cycles after stripping. The best flow rates seem to be 1 and 1.5 mL/min where late breakthrough points and the highest percentage of recovery were observed. However, the results obtained at

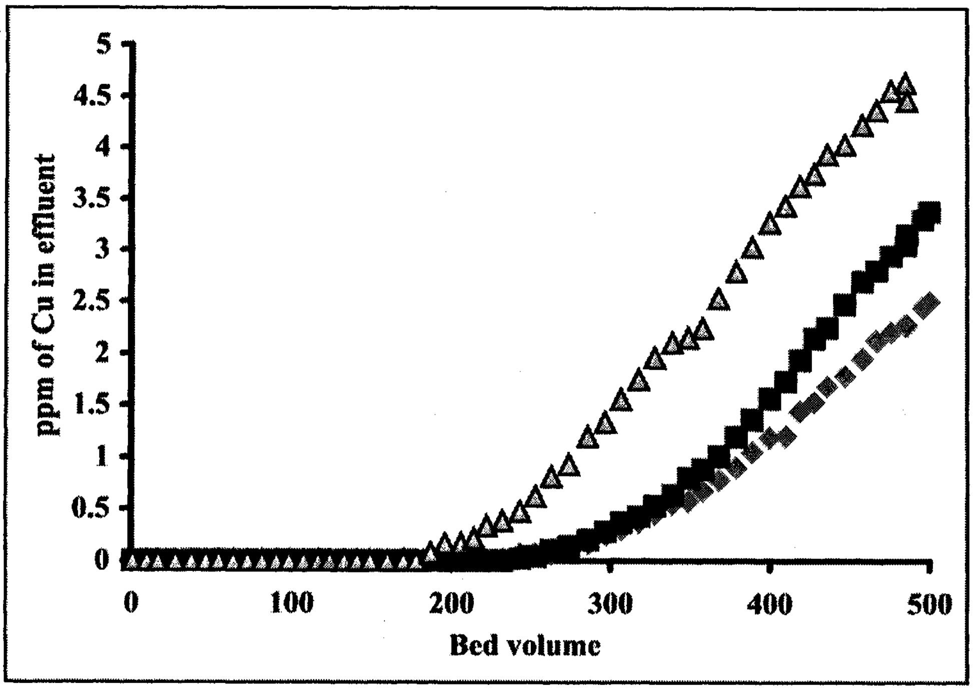

**Fig. 4:** Breakthrough curve for Cu(II) adsorption by humin biopolymer. Flow rate used was 3 mL/min. The 0.1mM Cu(II) solution was adjusted at pH 5 ± 0.1.
◆ 1st cycle, ■ 2nd cycle, ▲ 3rd cycle.

the flow rate of 2 mL/min were similar to those obtained at 1.5 mL/min. The preferential flow rate order for Cu binding to the immobilized-humin was 1mL/min>1.5mL/min≥2mL/min>3mL/min. In general, it was observed that the percentage of Cu recovered was pretty much the same in the three cycles, independently of the flow rate and the Cu binding as well. These percentages of recovery indicated that HCl is a good stripping agent for Cu. Furthermore, after three cycles, no changes in the Cu binding capacity to humin-silica matrix were observed.

### 3.2 Batch experiments for Ca(II) and Mg(II) interference on Cu(II) binding to silica-immobilized humin

The individual and combined effect of Ca(II) and Mg(II) on Cu(II) binding to silica-immobilized humin are shown in Figure 5. As it can be seen in the figure, Ca(II) and Mg(II) at concentrations of 2.0 mM and below did not interfere in the Cu binding to the humin biopolymer. Figure 5 also shows that Ca(II) interfered more than Mg(II). At the concentration of 200 mM, Ca(II) caused higher interference (66 %) than when Mg(II) was also present (69 %). Finally, at 1000 mM, the Cu(II) binding was 47% when Ca(II) was present, 44 % on the presence of Mg(II), and when both cations were present, the Cu binding dropped to 31 %. This might indicate that it is the quantity of the hard cations present in solution instead of their nature that contributes to the interference of Cu(II) binding to silica-immobilized humin. In previous studies, other

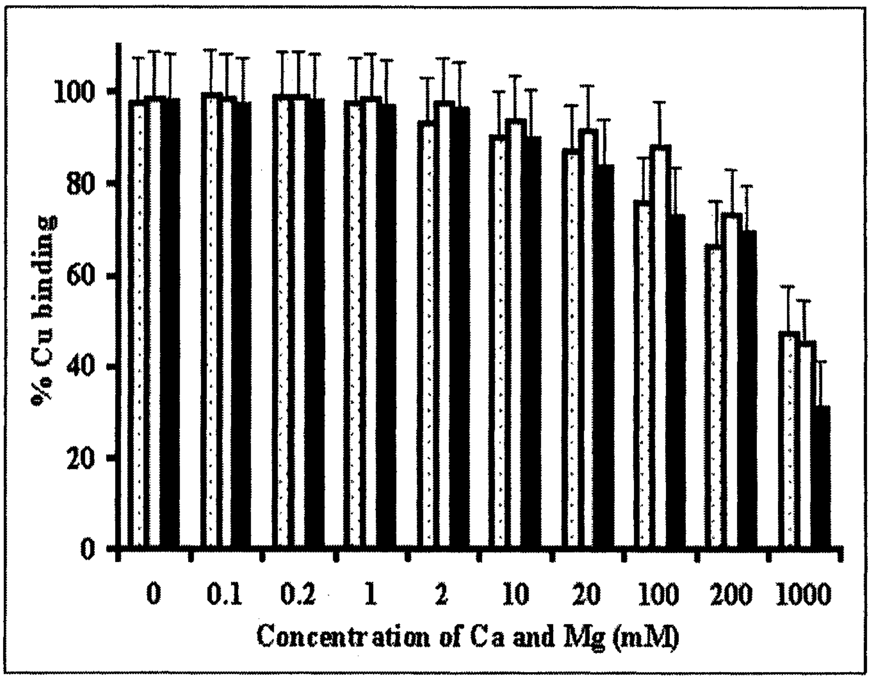

**Fig. 5:** Batch experiments for the interference of Ca(II) and Mg(II) on Cu(II) binding to humin biomass. Cu(II) concentration was kept constant at 0.1mM and the solution was adjusted to pH 5 ± 0.1; ⬚ Ca-Cu, □ Mg-Cu, ■ Ca-Mg/Cu. Error bars represent 95% C.I.

researchers found similar results using different biomasses /8,11/. These researchers concluded that the binding of different heavy metals to the functional groups present in the biomass may occur through an ion exchange reaction. Thus, the quantity of ions in solution is an important factor, instead of the competition for binding sites. Furthermore, Martell and Smith /15/ have proposed that some functional groups, such as carboxylates, have larger stability constants for heavy metals than for hard ions.

### 3.3 Column experiments for Ca(II) and Mg(II) interference on Cu binding to the humin biopolymer.

Column experiments were performed in order to determine the interference of Ca(II) and Mg(II) on Cu(II) binding to the silica-immobilized biopolymer under flow conditions. These experiments were carried out using individual and combined solutions of 1mM Ca(II) and Mg(II), a Cu(II) concentration of 0.1 mM, and a flow rate of 2mL/min. These parameters were chosen based on the data obtained in the batch experiments previously discussed. In addition, at the 2 mL/min flow rate, the binding capacity and Cu recovery was as good as at 1 mL/min flow rate, and time can be saved using the faster feasible velocity.

The breakthrough curves for the mixtures Cu-Ca, Cu-Mg, and Cu-Ca-Mg are shown in Figure 6. As one can see in this figure, in the presence of Mg(II), Cu appeared in the effluent after 250 bed volumes. However, when Ca(II) was present in the solutions, Cu appeared before 50 bed volumes, which indicates that Mg(II) slightly affected the Cu(II) binding to the humin biopolymer. Also, in the presence of Ca, after 500 bed

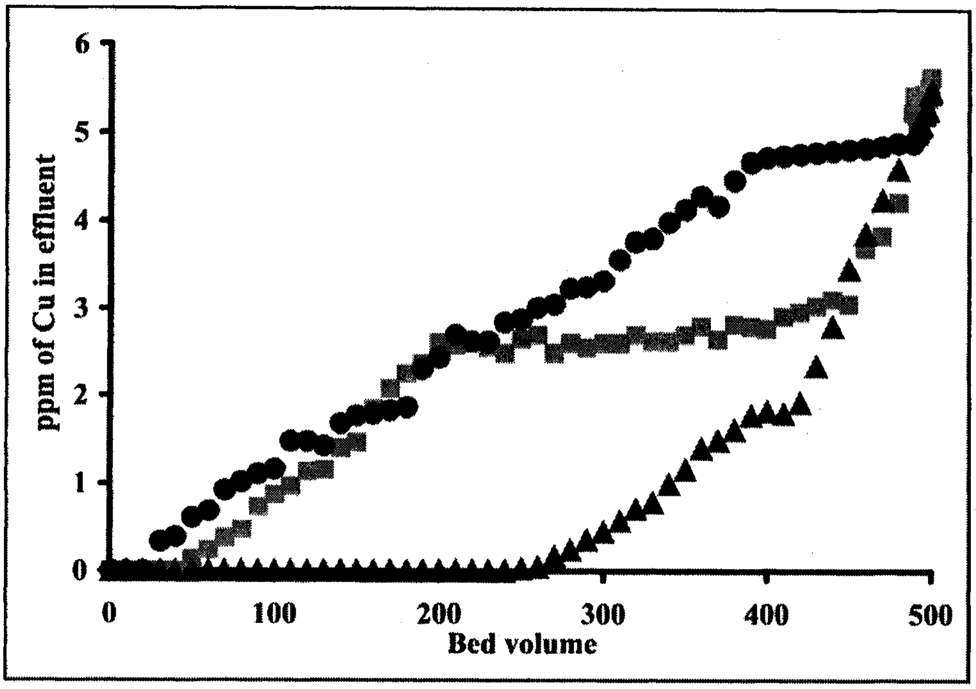

**Fig. 6:** Interference breakthrough curve on Cu(II) binding by humin immobilized-silica at a flow rate of 2 mL/min. The 0.1 mM Cu(II), 1 mM Mg(II), 1 mM Ca(II) solutions were adjusted to pH 5 ± 0.1; -■-Ca/Cu, -▲-Mg/Cu, -●-Ca-Mg/Cu volumes the column was not saturated, since at that point the Cu concentration in the effluent was 5.5 mg/L. This observation indicated that most of the interference for Cu(II) binding was due to Ca(II). The results indicate that the presence of hard cations at high concentrations interfere in the Cu binding to silica-immobilized humin. However, it is important to consider that the concentrations of Ca(II) and Mg(II) used in the column experiments were ten times higher than the concentration of Cu(II). Comparing Figure 6 and Figures 1-4, it can be seen that high concentrations of Ca(II) and Mg(II) strongly affected the Cu(II) binding capacity of the humin biopolymer.

### 3.4 Environmental Scanning Electron Microscopy (ESEM)

A scanning electron microphotograph of a small bead of silica-humin matrix after saturation with Cu is shown in Figure 7a. In this figure, bright dots can be observed. The mapping of the bead surface showed that the dots correspond to the signal for Cu (data not shown). These dots are between 923 nm to 2.16 μm in diameter. Figure 7b displays the energy dispersive X-ray spectroscopy (EDS) graph corresponding to Figure 7a. This figure clearly shows the peaks corresponding to Cu sorbed into the silica-polymer matrix (Si and Cu K emission signals are observed).

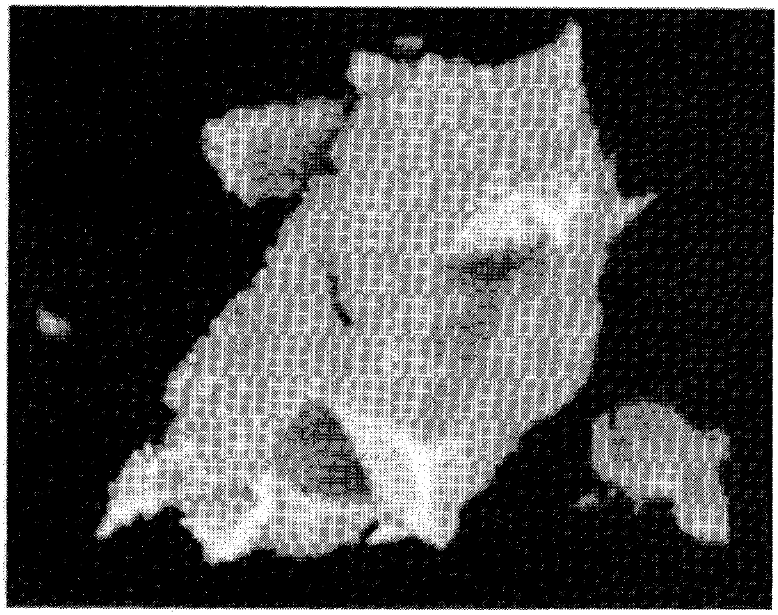

**Fig. 7a:** Scanning electron microscopy (SEM) image of the surface of silica-immobilized humin after saturation with copper. The bright dots represent the Cu(II) bound to the biopolymer.

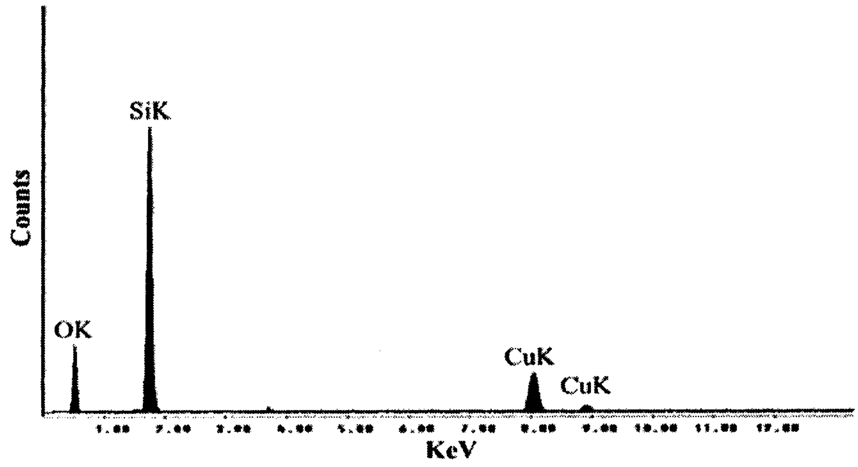

**Fig. 7b:** Energy dispersive spectrum (EDS) of Si and Cu observed in the surface of the humin biopolymer.

### 4. CONCLUSIONS

The results of this research showed that silica-immobilized humin is a promising alternative for Cu removal under various flow conditions. In the absence of hard cations, the breakthrough points for Cu(II) binding in a solution of pH 5 ± 0.1 were approximately 420, 390, 385, and 300 bed volumes at flow rates of 1, 1.5, 2 and 3 mL/min, respectively. The copper binding capacity of humin biopolymer was around 12 ± 1.5

mg/g and the percentages of Cu recovery had an average of 96.5% ± 1.5. At lower concentrations, e.g. less than 2.0 mM, Ca(II) and Mg(II) did not interfere with Cu(II) binding to the humin biopolymer. This suggests that silica-immobilized humin could be used as an inexpensive bio-sorbent for Cu removal from contaminated water.

### ACKNOWLEDGEMENTS

The authors acknowledge the financial support of the National Institute of Health (Grant S06GM8012-33). The authors would also like to acknowledge the financial support from the University of Texas at El Paso's Center for Environmental Resource Management (CERM) through funding from the Office of Exploratory Research of the EPA (Cooperative Agreement CR-819849-01-04). The authors also acknowledge the HBCU/MI Environmental Technology Consortium that is funded by the Department of Energy. Dr. Gardea-Torresdey acknowledges the funding from National Institute of Environmental Health Sciences (Grant R01ES11367-01). Guadalupe de la Rosa also acknowledges CONACyT (Consejo Nacional de Ciencia y Tecnología de México) (Grant # 131996)

### REFERENCES

1. F.J. Stevenson, *Humus Chemistry: Genesis, Composition, and Reactions.* John Wiley and Sons, New York, 1982.
2. G.R. Aiken, D.M. Mcknight, and R.L. Smith, *Humic Substances in Soil,Sediments and Water: Geochemistry, Isolation and Characterization.* Wiley-Interscience, New York, 1985; pp. 58, 276, 283.
3. D.L. Sparks, *Environmental Soil Chemistry.* San Diego, Academic Press, USA, 1995; pp. 70, 75, 78.
4. D.C. Sharma and C.F. Foster, *Biores. Technol.,* **52,** 261 (1995).
5. E. S. Bailey, J. T. Olin, R M. Brick, and D. D. Adrian, *Water Res.*, **33,** 2469 (1999)
6. G. de la Rosa, J.R. Peralta-Videa and J.L.Gardea-Torresdey, *J. Hazard. Mater.*, **97,** 207 (2003).
7. K. Kadirvelu, M. Palanival, R. Kalpana and S. Rajeswari, *Biores. Technol.,* **74,** 263 (2000).
8. J.L. Gardea-Torresdey, K.J. Tiemann, J.H. Gonzalez, J.A. Henning and M.S. Townsend, *J. Hazard. Mater.*, **48,** 181 (1996).
9. Y. Sag and Y. Aktay, *Process Biochem.*, **36,** 1187 (2001).
10. C. Namasivayam and K. Kadirvelu, *Chemosphere*, **34,** 377 (1997).
11. M. Spinti and H. Zhuang, *Water Environ. Res.*, **67,** 943 (1995).
12. Y.S. Ho and G. Mckay, *Water Res.*, **34,** 735 (2000).
13. J.L. Gardea-Torresdey, L. Tang and J.M. Salvador, *J. Hazard. Mater.*, **48.** 191 (1996).
14. D.A. Skoog and D.M. West, *Analytical Chemistry – an Introduction.* Saunders College Publishing, Philadelphia, 1994.
15. A.E. Martell and R.M. Smith, *Critical Stability Constants. Other Organic Ligands* Plenum Press, New York and London, 1997.

16. I.H. Segel, *Biochemical Calculations* 2nd Ed. Wiley, New York, 1976.
17. A. Kappor, T. Viraraghuan and D.R. Cullimore, *Biores. Technol.*, **70,** 95 (1999).
18. A. Blanco, B. Sanz and M.J. Llama, *J. Biotechnol.*, **69,** 227 (1999).
19. G. de la Rosa, J.L. Gardea-Torresdey, J.R. Peralta-Videa, I. Herrera and C. Contreras, *Biores. Technol.*, **90,** 11 (2003).
20. P.D. Johnson, M.A. Watson, J. Brown and L.A. Jefcoat, *Waste Manage.*, **22,** 471 (2002).

